# Is admittance to specialised palliative care among cancer patients related to sex, age and cancer diagnosis? A nation-wide study from the Danish Palliative Care Database (DPD)

**DOI:** 10.1186/s12904-017-0194-z

**Published:** 2017-03-23

**Authors:** Mathilde Adsersen, Lau Caspar Thygesen, Anders Bonde Jensen, Mette Asbjoern Neergaard, Per Sjøgren, Mogens Groenvold

**Affiliations:** 1 Research Unit, Department of Palliative Medicine, Bispebjerg Hospital, University of Copenhagen, 20D, Bispebjerg Bakke 23, Copenhagen, NV 2400 Denmark; 2 National Institute of Public Health, University of Southern Denmark, Copenhagen, Denmark; 3 Department of Oncology, Aarhus University Hospital, Aarhus, Denmark; 4 The Palliative Team, Department of Oncology, Aarhus University Hospital, Aarhus, Denmark; 5 Section of Palliative Medicine, Department of Oncology, Rigshospitalet, Copenhagen University Hospital, Copenhagen, Denmark; 6 Department of Public Health, University of Copenhagen, Copenhagen, Denmark

**Keywords:** Specialised palliative care, Cancer, End of life care, Hospice, Palliative care team, Admittance

## Abstract

**Background:**

Specialised palliative care (SPC) takes place in specialised services for patients with complex symptoms and problems. Little is known about what determines the admission of patients to SPC and whether there are differences in relation to institution type. The aims of the study were to investigate whether cancer patients’ admittance to SPC in Denmark varied in relation to sex, age and diagnosis, and whether the patterns differed by type of institution (hospital-based palliative care team/unit, hospice, or both).

**Methods:**

This was a register-based study of adult patients living in Denmark who died from cancer in 2010–2012. Data sources were the Danish Palliative Care Database, Danish Register of Causes of Death and Danish Cancer Registry. The associations between the explanatory variables (sex, age, diagnosis) and admittance to SPC were investigated using logistic regression.

**Results:**

In the study population (*N* = 44,548) the overall admittance proportion to SPC was 37%. Higher odds of overall admittance to SPC were found for women (OR = 1.23; 1.17–1.28), younger patients (<40 compared with 80+ years old) (OR = 6.44; 5.19–7.99) and patients with sarcoma, pancreatic and stomach cancers, whereas the lowest were for patients with haematological malignancies. The higher admission found for women was most pronounced for hospices compared to hospital-based palliative care teams/units, whereas higher admission of younger patients was more pronounced for hospital-based palliative care teams/units. Patients with brain cancer were more often admitted to hospices, whereas patients with prostate cancer were more often admitted to hospital-based palliative care teams/units.

**Conclusion:**

It is unlikely that the variations in relation to sex, age and cancer diagnoses can be fully explained by differences in need. Future research should investigate whether the groups having the lowest admittance to SPC receive sufficient palliative care elsewhere.

## Background

Specialised palliative care (SPC) takes place in specialised services for patients with complex symptoms and problems [1] and the majority of patients admitted to SPC worldwide and in Denmark have cancer diagnoses (about 95% in Denmark) [2, 3]. In Denmark SPC takes place in hospital-based palliative care teams/units and in hospices, but there are no national guidelines for referral of patients to SPC. Little is known about what determines the admission of patients to SPC [4] and whether there are differences in relation to institution type (hospital-based palliative care teams/units versus hospices).

Differences in symptoms and problems could explain differences in admittance to SPC. In a Danish nationally representative sample of patients with advanced cancer, it was shown that the majority of symptoms and problems were not associated with sex. The only differences reported were that patients with lung cancer had more symptoms and problems than patients with other cancer diagnoses and that older patients had more symptoms and problems compared with younger patients [5, 6].

In an optimally functioning health care system, admittance to SPC would be directly related to the level of needs. Other explanations could be differences in awareness, traditions and attitudes among the referring departments and SPC units. For example, there could be differences in the capacity among hospital departments and thereby in the incentive to refer patients to SPC. If such factors determine the referral, it may result in inequality (i.e., that patients with the same needs have different likelihood of admission) in admittance to SPC.

From the results of the previously mentioned Danish study of symptoms and problems among patients with advanced cancer, we have no reason to believe that certain subgroups defined by sex, age or cancer diagnosis will have higher needs for SPC; exceptions may be older persons and patients with lung cancer since these two groups had more symptoms [5, 6].

The aims of the present study were to investigate whether overall admittance to SPC in Denmark for adults who died from cancer varied with sex, age and cancer diagnosis, and whether the (admittance) patterns were different according to type of institution (admission to hospital-based palliative care teams/units, hospices, or both types of institutions).

## Methods

This is a Danish nation-wide register-based study. The unique Danish personal identification number (CPR-number) makes it possible to collect and merge data from different registers.

### Setting

There are two types of SPC institutions in Denmark (5.7 million inhabitants) but the characteristics of these are not mutually exclusive. First, there are hospices, and some of these also have a home care team (four out of 18 hospices had a home care team in addition to their in-patient facility). Hospices are free-standing services, separate from the rest of the health care system, and are publicly financed with no payment from patients. Secondly, there are hospital-based palliative care teams/units with or without an in-patient unit (five out of 26 hospital-based palliative care teams/units have an in-patient unit in addition to their home care and out-patient services). The teams/units are placed at hospitals, and are also fully publicly financed. During the study period, the number of SPC units in Denmark increased from 36 to 44 units [2].

About 80% of the patients admitted to SPC had contact with only one SPC unit (either a hospice or a hospital-based palliative care team/unit), whereas about 20% of the patients had contact with two or more SPC units, typically a hospital-based palliative care team followed by a hospice. All of these SPC units are expected to have multidisciplinary teams and to have weekly multidisciplinary meetings about their patients. The median survival time from the first referral to SPC to death was 27 days (mean 55 days) for patients admitted to a hospice, and for those admitted to a hospital-based palliative care team/unit it was 50 days (mean 90 days) [7].

### Data sources and variables

Danish Palliative Care Database (DPD) is a national quality of care database, and since 1 January 2010, it has been mandatory for all SPC units in Denmark to register all referred patients in the DPD. The DPD data about admission is validated against the Danish National Patient Register [8] in close collaboration with the SPC units. DPD has a high patient completeness, which has increased from 95.7% in 2010 [9] to 100% in 2012 [2]. Information about sex, age at the time of death, admittance to SPC (hospital-based palliative care team/unit and/or hospice) was collected from DPD.

Deaths among individuals living in Denmark are registered in Danish Register of Causes of Death (RCD). Information on cause of death is reported by the physician certifying the death. More than 99% of the death certificates contain complete data [10]. Data about the underlying cause of death (diagnosis) and date of death were obtained from RCD.

The Danish Cancer Registry (CR) is a population-based research register and contains incident cancer diagnoses since 1943. From 1987 it has been mandatory to report to CR [11]. CR contributed with information about cancer diagnoses.

Variables:

- *Overall admittance:* a dichotomous (yes/no) variable defined as any personal contact with SPC (inpatient, home visit, outpatient or palliative care team visits to inpatients at non-SPC departments). For patients with more than one contact the information from the first contact was included.
- *Institution type specific admittance:* Overall admittance subdivided after type, i.e., hospital-based palliative care team/unit, hospice or both.
- *Explanatory variables:* sex, age at the time of death grouped as: 18–39, 40–49, 50–59, 60–69, 70–79, 80+ years, and cancer diagnosis coded using ICD-10 (Table 1).

**Table 1:** The characteristics of the study population overall and institution type specific admittance

	N	Overall admittance to SPC	Admittance to hospital-based palliative care team/unit	Admittance to Hospice	Admittance to both hospital-based palliative care team/unit and hospice
	(%)	%	%	%	%
All cancer diagnoses	44,548 (100)	37.4	26.8	17.3	6.8
Sex					
Men	23,312 (52.3)	35.5	26.8	14.7	6.0
Women	21,236 (47.7)	39.5	26.8	20.2	7.8
Age (years)					
18–39	406 (0.9)	65.0	52.2	30.5	17.7
40–49	1,353 (3.0)	59.7	46.3	29.3	16.0
50–59	4,521 (10.2)	51.1	39.2	22.8	10.8
60–69	11,221 (25.2)	44.3	32.5	20.3	8.5
70–79	13,870 (31.1)	36.7	25.9	16.9	6.1
80+	13,177 (29.6)	24.3	15.9	11.7	3.3
Diagnosis (cancer site)					
Oral cavity, nasopharyngeal (etc.) (C00-C14)	998 (2.2)	37.7	30.0	16.2	8.5
Oesophageal (C15)	1,101 (2.5)	43.0	32.5	17.3	6.8
Stomach (C16)	1,285 (2.9)	47.6	35.4	22.0	9.9
Small intestine (C17)	162 (0.4)	40.1	30.9	17.3	8.0
Colorectal (C18–C20)	5,649 (12.7)	36.0	25.9	16.7	6.6
Liver (etc.) (C22)	845 (1.9)	34.7	24.6	14.0	3.9
Pancreatic (C25)	2,473 (5.6)	49.4	34.9	22.6	8.1
Laryngeal (C32)	280 (0.6)	29.6	22.5	12.9	5.7
Tracheal, bronchial and lung (C33–C34)	10,338 (23.2)	39.5	28.2	18.4	7.1
Melanoma skin cancer (C43)	774 (1.7)	45.1	31.7	23.1	9.7
Sarcoma (C46–C49)	326 (0.7)	54.9	40.8	25.8	11.7
Breast (C50)	3,618 (8.1)	37.3	25.9	18.3	6.9
Cervical (C53)	301 (0.7)	45.2	31.6	26.3	12.6
Uterine (C54–55)	499 (1.1)	42.1	29.1	20.2	7.2
Ovarian (etc.) (C56,C570–C574)	1,109 (2.5)	49.9	33.3	27.7	11.1
Prostate (C61)	3,512 (7.9)	33.9	27.7	12.6	6.4
Testicular (C62)	39 (0.1)	30.8	28.2	12.8	10.3
Kidney (etc.) (C64–C66)	991 (2.2)	44.4	33.5	18.8	7.9
Bladder (C67)	1,378 (3.1)	32.3	24.2	13.4	5.4
Brain/central nervous system (C70–C71, C751–C753)^a^	1,407 (3.2)	40.9	26.0	22.3	7.4
Thyroid (C73)	114 (0.3)	45.6	32.5	21.1	7.9
Unknown primary cancer (C76–C80)	1,802 (4.1)	32.4	21.6	15.8	5.1
Hodgkin disease (C81)	58 (0.1)	19.0	13.8	10.3	5.2
Non-Hodgkin lymphoma (C82-C85)	814 (1.8)	20.2	11.7	11.4	3.0
Multiple myeloma (C 90)	635 (1.4)	20.6	14.0	9.3	2.7
Leukemia (C91–C95)	1,200 (2.7)	15.3	8.4	7.9	1.1
Other cancer (all other C codes)	2,840 (6.4)	29.9	21.4	13.8	5.3

^a^Including the following D-codes: D32, D42, D330–332, D352–354, D430–432, D443–445, D333–339 and D433–439

### Population/sample

**Fig. 1:**
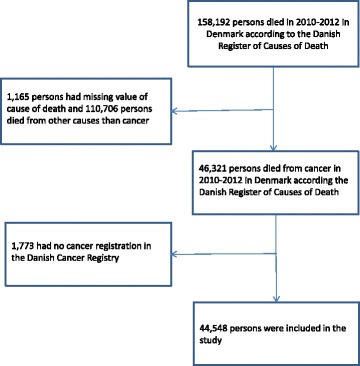
Flow-chart for sampling the study population

From RCD we identified adults (at least 18 years old) with cancer as the underlying cause of death in 2010–2012 including all ICD-10 C-codes and the D-codes for cancers in the brain (see Table 1). The death causes of cancer were validated against the CR [11]: (i) For most patients (84%) the same diagnosis was found in the two registers. (ii) Different diagnoses were found for 12%, these individuals were included in the study with the cancer diagnosis registered in CR. If there was more than one cancer registration, the latest was used. (iii) Patients with no cancer diagnosis registered in the CR were excluded (4%, *N* = 1,773). After these exclusions 44,548 patients were included in the study (Fig. 1).

### Data analysis

The associations between the explanatory variables (sex, age and cancer diagnosis) and overall admittance to SPC were investigated using unadjusted and adjusted logistic regression analysis including all the explanatory variables in the model. In addition, three separate multiple logistic regression analyses were made for admittance to hospital-based palliative care team/unit, hospice or both, respectively, adjusted for sex, age and diagnosis. The reference group for diagnosis was the average of admittance for all diagnoses. The results from the logistic regressions are reported as odds ratios (ORs) with 95% confidence intervals (CIs). As level of statistical significance *p* < 0.05 was used. The analyses were carried out using SAS statistical software version 9.3 [12].

## Results

In the study population (*N* = 44,548), the overall admittance proportion to SPC was 37.4% of all patients dying of cancer in 2010–12. The institution type specific admittance was 26.8% for hospital-based palliative care team/unit and 17.3% for hospice. Thus, some patients (6.8%) were admitted to hospital-based palliative care team/unit and hospice (Table 1).

### Sex

In the study population slightly more than half were men (52.3%). Overall, women had a higher admittance proportion (39.5%) than men (35.5%) (Table 1).

The multiple logistic regression analysis showed that overall, women were more likely to be admitted to SPC than men (OR = 1.23; 95% CI:1.17–1.28) (Table 2). The institution type specific admittance analyses showed that this sex difference was more pronounced in relation to hospice (OR = 1.45; 95% CI:1.37–1.54), than for hospital-based palliative care team/unit (OR = 1.06; 95% CI:1.00–1.11).

**Table 2:** Overall and institution type specific odds of admittance to SPC for Danish cancer patients, mutually adjusted

	Overall admittance to SPC	Admittance to hospital-based palliative care team/unit	Admittance to hospice	Admittance to both hospital-based palliative care team/unit and hospice
	OR (95% CI)	OR (95% CI)	OR (95% CI)	OR (95% CI)
Sex	(*P* < 0.001)	(*P* < 0.033)	(*P* < 0.001)	(*P* < 0.001)
Women	1.23 (1.17–1.28)	1.06 (1.00–1.11)	1.45 (1.37–1.54)	1.34 (1.23–1.47)
Men	1 (ref)	1 (ref)	1 (ref)	1 (ref)
Age (years)	(*P* < 0.001)	(*P* < 0.001)	(*P* < 0.001)	(*P* < 0.001)
18–39	6.44 (5.19–7.99)	6.81 (5.53–8.38)	3.17 (2.54–3.97)	6.73 (5.08–8.93)
40–49	4.60 (4.09–5.18)	4.80 (4.26–5.41)	2.90 (2.54–3.31)	5.64 (4.72–6.74)
50–59	3.22 (3.00–3.47)	3.48 (3.22–3.76)	2.13 (1.94–2.32)	3.63 (3.16–4.17)
60–69	2.46 (2.32–2.60)	2.56 (2.41–2.73)	1.89 (1.76–2.03)	2.80 (2.49–3.16)
70–79	1.80 (1.70–1.89)	1.86 (1.75–1.97)	1.52 (1.42–1.64)	1.97 (1.74–2.22)
80+	1 (ref)	1 (ref)	1 (ref)	1 (ref)
Diagnosis (cancer site)	(*P* < 0.001)	(*P* < 0.001)	(*P* < 0.001)	(*P* < 0.001)
Oral cavity, nasopharyngeal (etc.) (C00–C14)	0.95 (0.83–1.09)	1.07 (0.93–1.23)	0.94 (0.79–1.12)	1.23 (0.97–1.55)
Oesophageal (C15)	1.34 (1.18–1.52)	1.36 (1.19–1.56)	1.09 (0.93–1.29)	1.08 (0.85–1.38)
Stomach (C16)	1.69 (1.50–1.90)	1.63 (1.44–1.85)	1.50 (1.30–1.73)	1.67 (1.37–2.04)
Small intestine (C17)	1.23 (0.89–1.68)	1.34 (0.96–1.87)	1.07 (0.72–1.59)	1.31 (0.75–2.29)
Colorectal (C18–C20)	1.13 (1.05–1.22)	1.16 (1.07–1.26)	1.09 (0.99–1.19)	1.18 (1.03–1.35)
Liver (etc.) (C22)	0.92 (0.80–1.07)	0.91 (0.77–1.07)	0.83 (0.68–1.02)	0.59 (0.42–0.84)
Pancreatic (C25)	1.77 (1.61–1.94)	1.59 (1.44–1.75)	1.47 (1.31–1.64)	1.31 (1.11–1.54)
Laryngeal (C32)	0.75 (0.58–0.97)	0.82 (0.62–1.08)	0.80 (0.57–1.13)	0.93 (0.57–1.53)
Tracheal, bronchial and lung (C33–C34)	1.16 (1.09–1.23)	1.15 (1.07–1.23)	1.12 (1.03–1.21)	1.12 (1.00–1.25)
Melanoma skin cancer (C43)	1.46 (1.26–1.69)	1.31 (1.12–1.54)	1.52 (1.28–1.81)	1.53 (1.19–1.96)
Sarcoma (C46–C49)	1.90 (1.52–2.38)	1.74 (1.39–2.19)	1.54 (1.20–1.98)	1.58 (1.12–2.23)
Breast (C50)	0.96 (0.88–1.05)	0.99 (0.90–1.09)	0.94 (0.84–1.04)	0.93 (0.79–1.08)
Cervical (C53)	1.10 (0.87–1.39)	1.06 (0.83–1.36)	1.30 (1.00–1.69)	1.43 (1.01–2.03)
Uterine (C54–55)	1.35 (1.13–1.62)	1.36 (1.11–1.66)	1.16 (0.93–1.45)	1.15 (0.82–1.62)
Ovarian (etc.) (C56,C570–C574)	1.57 (1.38–1.78)	1.38 (1.20–1.58)	1.56 (1.35–1.80)	1.52 (1.23–1.86)
Prostate (C61)	1.32 (1.21–1.44)	1.54 (1.40–1.69)	1.04 (0.93–1.18)	1.58 (1.34–1.87)
Testicular (C62)	0.50 (0.26–0.99)	0.68 (0.34–1.37)	0.63 (0.25–1.56)	1.11 (0.40–3.06)
Kidney (etc.) (C64–C66)	1.46 (1.28–1.66)	1.49 (1.30–1.71)	1.19 (1.01–1.40)	1.28 (1.01–1.63)
Bladder (C67)	1.04 (0.92–1.17)	1.13 (0.99–1.29)	0.92 (0.78–1.08)	1.06 (0.83–1.35)
Brain/CNS (C70–C71, C751–C753)^a^	1.05 (0.98–1.17)	0.85 (0.75–0.97)	1.30 (1.13–1.49)	0.97 (0.78–1.20)
Thyroid (C73)	1.54 (1.07–2.22)	1.49 (1.01–2.20)	1.29 (0.83–2.00)	1.25 (0.64–2.43)
Unknown primary cancer (C76–C80)	0.93 (0.83–1.04)	0.89 (0.79–1.00)	0.99 (0.86–1.13)	0.86 (0.69–1.07)
Hodgkin disease (C81)	0.33 (0.17–0.63)	0.37 (0.18–0.77)	0.52 (0.23–1.17)	0.65 (0.21–2.03)
Non-Hodgkin lymphoma (C82–C85)	0.49 (0.42–0.60)	0.41 (0.33–0.51)	0.70 (0.56–0.87)	0.50 (0.33–0.74)
Multiple myeloma (C 90)	0.50 (0.42–0.61)	0.52 (0.42–0.65)	0.55 (0.42–0.72)	0.46 (0.28–0.73)
Leukemia (C91–C95)	0.34 (0.29–0.40)	0.29 (0.23–0.35)	0.47 (0.38–0.58)	0.18 (0.10–0.30)
Other cancer (all other C codes)	0.91 (0.83–1.00)	0.96 (0.87–1.07)	0.90 (0,80–1,02)	0.99 (0,83–1,19)
Average of all diagnoses	1 (ref)	1 (ref)	1 (ref)	1 (ref)

^a^Including the following D-codes: D32, D42, D330–332, D352–354, D430–432, D443–445, D333–339 and D433–439

### Age

Most patients were above the age of 60 years (85%), while only 0.9% were 18–39 years old (Table 1). A much higher admittance proportion to SPC was found for younger compared with older patients; the overall admittance proportion decreased from 65.0% for individuals 18–39 years of age to 24.3% for those age 80+ years old (Table 1).

In the multiple logistic regression analysis a strong association between age and overall admittance was found (Table 2). The odds of admittance to SPC were over six times higher for the youngest (18–39 years old) compared to the 80+ years old (OR = 6.44; 95% CI:5.19–7.99). Looking at institution type specific admittance, the differences between age groups were more pronounced for admittance to hospital-based palliative care team/unit than for hospice.

### Diagnosis

The most common cancer diagnoses in the study population were lung (23.2%), colorectal (12.7%) and breast cancer (8.1%) (Table 1).

The highest odds of overall admittance to SPC were found for individuals with sarcoma (OR = 1.90; 95% CI:1.52–2.38), pancreatic (OR = 1.77; 95% CI:1.61–1.94) and stomach cancer (OR = 1.69; 95% CI:1.50–1.90) compared with the average of all diagnoses (Table 2). The lowest odds of overall admittance to SPC were found for patients with haematological malignancies with odds ratios between 0.33 (95% CI:0.17–0.63) and 0.50 (95% CI:0.42–0.61).

The multiple logistic regression analyses of the institution type specific admittance showed a somewhat different pattern in relation to diagnosis. The admittance to hospital-based palliative care team/unit was consistent with the overall admittance to SPC. High admittance to hospice was also found in relation to patients with sarcoma cancer (OR = 1.54; 95% CI:1.20–1.98), but the highest odds were for patients with ovarian cancer (OR = 1.56; 95% CI:1.35–1.80). Again the lowest odds of admittance were found for patients with haematological malignancies in relation to all types of institutions. For patients with some cancer diagnoses large differences were seen between use of hospital-based palliative care team/unit and hospice: for patients with brain cancer the odds of admittance to hospice were markedly above the average of all diagnoses, whereas the odds of admittance to hospital-based palliative care teams/units were below the average of all diagnoses. In relation to patients with prostate cancer the reverse pattern was found.

## Discussion

We found relatively large differences in admittance to SPC: lower admittance to SPC for men (most pronounced for hospice than hospital-based palliative care team/unit), markedly lower admission for older patients (more pronounced for hospital-based palliative care team/unit than hospice) and lower admission for patients with haematological diseases. For two cancer diagnoses the patterns were opposite: there was higher admittance than average to hospice and lower admittance to hospital-based palliative care team/unit for patients with brain cancer whereas there was higher admittance to hospital-based palliative care team/unit and lower to hospice for patients with prostate cancer.

The study was based on data from well-established nation-wide registers with high completeness [2, 7, 9, 11, 13]. This ensured a large national study population (*N* = 44,548) of all patients who died of cancer in 2010–2012 in Denmark, which makes it possible to study the population in detail, e.g. looking at the different diagnoses separately. Further, it ensures representativeness and minimizes the effect of selection bias [14]. We have found no other studies of similar size and quality, and no previous studies have been comparing the patterns of admittance to different types of SPC institutions.

A high validity of the variable “admittance to SPC” from the DPD was ensured by validating the data of admittance from DPD against the Danish National Patient Register [8] and SPC institutions were contacted if uncertainties were present the. Furthermore, the cancer diagnoses registered in the Danish Register of Causes of Death were validated against the Danish Cancer Registry, which has high quality of data with 89% of the tumours being morphologically verified [11]. In the present study only 1,773 (4%) patients were excluded because the registration of the cancer diagnoses in the Danish Register of Causes of Death was not found in the Danish Cancer Registry. This ensured high validity of the diagnosis variable and with the very limited number of cases excluded one must expect only a minor influence on the results.

The results from the present study can be compared with the results from a large Danish study, described in the introduction, investigating symptoms and problems in patients with advanced cancer [5, 6].

We found that women were more often admitted to SPC, especially to hospice (OR 1.45; 1.37–1.54). The Danish study of needs [5, 6] showed only minor differences in symptoms and problems in relation to sex, although there was a tendency towards worse emotional function for women with solid tumours [5, 6]. Such a difference might contribute to the sex difference but it seems unlikely that it explains the marked disparity found in this study. Some earlier studies reported higher admittance to SPC for women [15–18], whereas most studies did not show any differences [3]. The sex difference found in Denmark could be explained by traditional sex roles where women to a larger extent than men provide end of life care at home to their partner. Other possible explanations could be that the needs of women are more compatible with hospice or that more women prefer the hospice option. It might also be that women are better at recognizing and articulating a need for hospice; this would be in line with findings that women are more likely to talk about their own impending death [19] and to acknowledge that their illness is incurable [20].

In the present study admittance to SPC decreased with increasing age, whereas the study of symptoms and problems found that symptoms and problems seemed to increase with increasing age [5, 6]. Our results are in line with earlier studies [21] although the age gradient was much stronger in our study [16, 22–25]. Comparing the youngest with the oldest patients a stronger association was found for hospital-based palliative care team/unit (OR = 6.81; 5.53–8.38) than for hospice (OR = 3.17; 2.57–3.97). With the limited SPC capacity and if we accept that the need is not lower among the oldest, some older patients may not receive the care they need, maybe because symptoms and death are more accepted in relation to older than younger patients. It is unknown whether other parts of the health care system compensate for the lower admission to SPC among the older patients, e.g., whether the needs are adequately covered by primary care, where they may already be in close contact with the general practitioner, or via nursing homes.

Comparing the overall admittance to SPC of patients with different cancer diagnoses, admittance was highest for patients with sarcoma, pancreatic and stomach cancers and lowest for patients with haematological malignancies. According to the previous study of advanced cancer patients the patterns for admittance cannot be explained by differences in needs: the patient groups having higher admittance did not have more symptoms and problems, and patients with haematological malignancies did not have fewer symptoms [5, 6]. However, there may of course be differences in needs that were not revealed in the questionnaires used. Previous studies have also found that, patients with haematological malignancies were less often admitted to SPC [26, 27]. It has also been reported that the patients with haematological cancer are referred closer to death than patients with other cancer diagnoses and receive more aggressive treatment towards the end of life [28–31]. The fact that active cancer treatment of this patient group continues until close to death may explain why these patients less often are admitted to SPC. It may be a wish from the patients to continue their trajectory at the haematological departments and not be referred to a new and unknown SPC unit. More research is needed to better understand the referral of patients with haematological malignancies to SPC. In relation to gynaecological and gastrointestinal cancer we found like Hui et al. higher odds of admittance to SPC [27], especially for ovarian, pancreatic and stomach cancers, but with marked variation between the different cancer types in each subgroup and between admittance to hospital-based palliative care team/unit and hospice. Other studies found lower admittance for breast cancer compared to colorectal cancer patients [22] and lung cancer patients [32], similar to the present study but different from the study by Hui et al. [27].

Differences in admittance in relation to type of institution were primarily found in relation to patients with brain and prostate cancer. Possibly, some types of cancer are more compatible with certain types of care than others. The care of patients with prostate cancer more frequently takes place in hospital-based palliative teams/units, and this may reflect that it can take place in the home of the patient. The care of brain cancer patients may be more demanding, with difficult symptoms such as cognitive impairment and personality changes, which may be more difficult to accommodate by the family caregivers, leading to hospice referral.

It is a recurring discussion in studies of admittance to SPC whether differences between subgroups reflect a real difference in need or an inequality, as the burden of symptoms and problems (reflecting the need) are unknown. It is a strength of this study that it has been possible to compare admittance to SPC against the pattern of symptoms and problems in a nationally sample covering all advanced cancer patient. Of course, the comparison has some limitations, for example the EORTC QLQ-C30 questionnaire, which was used may not cover all relevant aspects, for example the availability of help and social support at home.

Given the large differences in admittance in relation to sex, age and diagnosis, future research should investigate whether the groups having the lowest admittance have uncovered needs of SPC (e.g. via surveys of patients not admitted or by evaluating whether those admitted have more needs, indicating a higher degree of selection). It could also be relevant to compare end of life outcomes reported by bereaved carers. Finally, it would be relevant to compare the geographic patterns in more detail.

This study examines “overall admittance to SPC”. The DPD includes the date and type of initial contact with SPC but does not contain the detailed data about the frequency and nature of additional contacts with SPC. In an ongoing development project we are working on establishing such data from other registries but these data are not yet available. When such detailed data about the number and nature of SPC contacts become available, they can answer important additional research questions.

In this study SPC was separated into the two categories, hospital-based palliative care team/unit and hospice. This is a construction with limitations, because about 20% of the hospices have outgoing palliative teams and 20% of the hospital-based palliative care teams/units have SPC units with in-patients like hospices. On the other hand there are some clear differences between hospices and hospitals e.g. the organisation, financing and whether or not it takes place within the hospital system, which makes the distinction meaningful. However, due to the fact that each of the categories did include aspects from the ‘opposite’, our categorisation may underestimate the differences between hospital-based palliative care team/unit and hospice.

## Conclusion

In this first nation-wide register-based study of admittance to SPC among patients with cancer we found lower overall admittance for men, older patients and patients with haematological malignancies. Compared with hospital-based palliative care team/unit, admittance to hospice was lower for men and for patients with prostate cancer whereas admittance to hospice was higher for patients with brain cancers. The large variation in admittance to SPC found in this study in relation to sex, age and cancer types cannot be explained by the variation in symptoms and problems among advanced cancer patients in general. Future research should investigate whether the groups having the lowest admittance to SPC receive sufficient palliative care.

## Abbreviations

CR: The Danish Cancer Registry
DPD: Danish Palliative Care Database
OR: Odds ratio
RCD: Danish Register of Causes of Death
SPC: Specialised palliative care
